# DNA Damage-Induced Ferroptosis: A Boolean Model Regulating p53 and Non-Coding RNAs in Drug Resistance

**DOI:** 10.3390/proteomes13010006

**Published:** 2025-01-20

**Authors:** Shantanu Gupta, Daner A. Silveira, José Carlos M. Mombach, Ronaldo F. Hashimoto

**Affiliations:** 1 Instituto de Matemática e Estatística, Departamento de Ciência da Computação, Universidade de São Paulo, Rua do Matão 1010, São Paulo 05508-090, SP, Brazil; ronaldo@ime.usp.br; 2 Children’s Cancer Institute, Porto Alegre 90620-110, RS, Brazil; d.silveira.bioinfo@ici.ong; 3 Departamento de Física, Universidade Federal de Santa Maria, Santa Maria 97105-900, RS, Brazil; jcmombach@ufsm.br

**Keywords:** CricRNA NOTCH1, lncRNA MALAT1, miR-34c-5p, p53, apoptosis, ferroptosis

## Abstract

The tumor suppressor p53, in its wild-type form, plays a central role in cellular homeostasis by regulating senescence, apoptosis, and autophagy within the DNA damage response (DDR). Recent findings suggest that wild-type p53 also governs ferroptosis, an iron-dependent cell death process driven by lipid peroxidation. Post-translational modifications of p53 generate proteoforms that significantly enhance its functional diversity in regulating these mechanisms. A key target in this process is the cystine/glutamate transporter (xCT), which is essential for redox balance and ferroptosis resistance. Additionally, p53-induced miR-34c-5p suppresses cancer cell proliferation and drug resistance by modulating Myc, an oncogene further influenced by non-coding RNAs like circular RNA NOTCH1 (CricNOTCH1) and long non-coding RNA MALAT1. However, the exact role of these molecules in ferroptosis remains unclear. To address this, we introduce the first dynamic Boolean model that delineates the influence of these ncRNAs and p53 on ferroptosis, apoptosis, and senescence within the DDR context. Validated through gain- and loss-of-function perturbations, our model closely aligns with experimental observations in cancers such as oral squamous cell carcinoma, nasopharyngeal carcinoma, and osteosarcoma. The model identifies crucial positive feedback loops (CricNOTCH1/miR-34c/Myc, MALAT1/miR-34c/Myc, and Myc/xCT) and highlights the therapeutic potential of using p53 proteoforms and ncRNAs to combat drug resistance and induce cancer cell death.

## 1. Introduction

The tumor suppressor p53 is one of the most well-studied proteins in cancer biology and is known for its pivotal role in maintaining cellular homeostasis. As a key regulator of the DNA damage response (DDR), p53 orchestrates several cellular processes, including cell cycle arrest, apoptosis, senescence, and autophagy [1,2,3], ensuring that damaged cells either repair themselves or undergo programmed cell death. Proteoforms of p53, generated through post-translational modifications (PTMs) and mutations, play distinct roles in these processes, underscoring the functional complexity of the p53-regulated network [4]. Recent evidence suggests that it also plays a crucial role in ferroptosis, an iron-dependent form of cell death triggered by lipid peroxidation [5]. This novel mechanism opens new avenues for understanding how p53 proteoforms influence cell fate under stress conditions, particularly in cancer [5].

A major player in the ferroptosis pathway regulated by p53 is the cystine/glutamate transporter (xCT), which is essential for maintaining redox balance and resisting ferroptosis [5]. xCT facilitates the uptake of cystine and the export of glutamate, thereby regulating intracellular levels of glutathione, a critical antioxidant. By repressing xCT, specific proteoforms of p53 can lower the cell’s antioxidant defenses, increasing susceptibility to ferroptosis and enhancing the efficacy of cancer therapies [5]. Additionally, p53 activates Spermidine/Spermine N1-Acetyltransferase 1 (SAT1), which promotes lipid peroxidation, further driving ferroptosis. SAT1 induction is also linked to the expression of arachidonate 15-lipoxygenase (ALOX15), an enzyme that catalyzes lipid peroxidation, amplifying the ferroptotic process [6].

Myc is known to upregulate xCT expression [7,8]; at the same time, xCT also regulates Myc expression by increasing intracellular glutamate levels, which enhances Myc’s transcriptional activity [9,10], promoting both survival and drug resistance in cancer cells. This dynamic relationship between Myc and xCT highlights the complexity of p53’s regulatory network, where proteoform-specific functions of p53 may counteract the pro-survival effects of Myc, facilitating the emergence of a more resistant tumor phenotype [7,9].

Non-coding RNAs (ncRNAs), including circular RNAs (circRNAs), long non-coding RNAs (lncRNAs), and microRNAs (miRNAs), play crucial roles in gene expression regulation and cellular processes [11]. CircRNAs, with their closed loop structure, act as molecular sponges for miRNAs, modulating gene expression [11]. LncRNAs, which are typically over 200 nucleotides, are involved in chromatin remodeling and transcriptional regulation, influencing differentiation and stress responses. MiRNAs, which are about 22 nucleotides long, regulate gene expression post-transcriptionally by binding to target mRNAs, leading to their degradation or translational inhibition [11]. Together, these ncRNAs are essential for cellular homeostasis and have significant implications in diseases, particularly cancer [11]. In this context, ncRNAs such as circular RNA NOTCH1 (CricNOTCH1) and lncRNA Metastasis-Associated Lung Adenocarcinoma Transcript 1 (lncRNA MALAT1) further complicate the regulatory landscape. Both CricNOTCH1 and MALAT1 are upregulated by Myc and function as oncogenes [12,13], contributing to drug resistance by antagonizing specific proteoforms of p53 [14] and inhibiting microRNA-34c-5p (hereafter referred to as miR-34c) [12,15]. Recent studies indicate that these ncRNAs not only inhibit miR-34c but also enhance Myc signaling [12,15], establishing regulatory mechanisms that drive tumorigenesis and resistance to cell death pathways such as apoptosis and ferroptosis.

**Figure 1:**
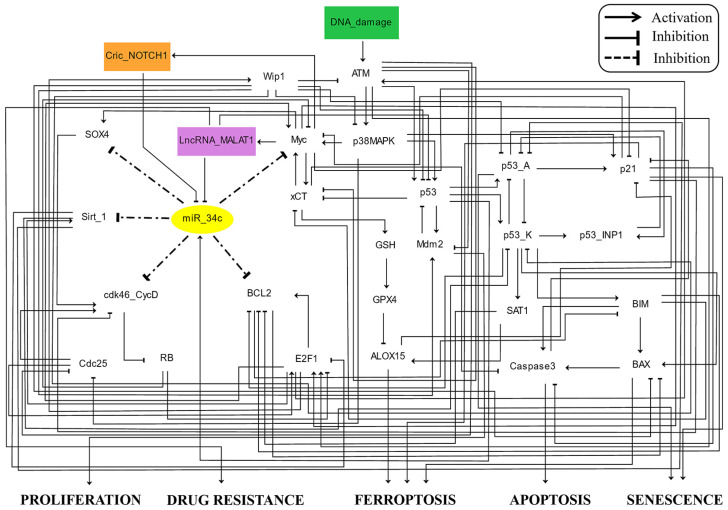
A dynamic Boolean model of ncRNAs and p53 in ferroptosis. Direct black edges ending in arrowheads represent positive regulatory interactions, while those ending in hammerheads indicate negative ones. Dashed black edges ending with hammerheads specify miR-34c targets. Node colors represent function: signaling proteins are white, CricNOTCH1 is an orange rectangle, and lncRNA MALAT1 is a purple rectangle, with miR-34c as a yellow oval. The green rectangle indicates DNA damage. Model outputs are labeled as proliferation, drug resistance, ferroptosis, and apoptosis.

The tumor suppressor p53 regulates critical cell fate decisions, including apoptosis, senescence, and ferroptosis, within the DDR. Our study presents a novel dynamic Boolean model (see Figure 1) that, to our knowledge, is the first of its kind to orchestrate the processes of ferroptosis, apoptosis, and senescence in cancer cells. By integrating key regulators such as CricNOTCH1, lncRNA MALAT1, miR-34c, Myc, and p53, the model elucidates the interplay among these pathways. Addressing redox homeostasis, proteome complexity, and oxidative stress, the model offers insights into leveraging ferroptosis as a redox-dependent form of cell death to overcome drug resistance in cancer therapy, emphasizing WTp53-driven mechanisms and the role of ncRNAs.

## 2. Materials and Methods

### 2.1. Navigating the Gene Regulatory Network Terrain in Cancer Cells: Merging Public Databases and Tools

We established a comprehensive gene regulatory framework to delve into the roles of CricNOTCH1, lncRNA MALAT1 and miR-34c in ferroptosis activation in DDR. Leveraging PubMed and BIOGRID 3.5 (https://thebiogrid.org/) [16], we meticulously explored the relevant literature and utilized GINsim 3.0.0b (http://www.ginsim.org/downloads) [17] to construct a Boolean model. To gain insights into the dynamic behavior of the model, we employed stochastic simulations with MaBoSS 2.0 (https://maboss.curie.fr/) [18]. The GINsim model file was seamlessly exported to MaBoSS, enabling the use of stochastic trajectory calculations to quantitatively assess component state probabilities. We maintained identical transition rates, utilized a time increment of 0.1, and conducted simulations over a maximum of 40 time steps to ensure robust analysis. For access to the model file, refer to the “Code Availability” section.

### 2.2. Constructing Dynamic Boolean Network Models, Formulating Rules, and Simulating PubMed-Inspired Insights

Boolean analysis examines a regulatory graph where nodes represent signaling components and edges indicate activation or inhibition. Each node operates as a Boolean variable, and is either “0” (inactive) or “1” (active). Logical rules, based on biochemical data, determine node activation.

To model CricNOTCH1, lncRNA MALAT1, and miR-34c in ferroptosis activation in DDR, we translated gene interactions into Boolean rules, as detailed in Table S1. Using basic Boolean operators like “AND”, “OR”, and “NOT”, we formulated these rules. Simulations with the Boolean network produce attractors, and a state transition graph (STG) illustrates model dynamics. In the STG, nodes represent network states with arcs showing transitions. Stable states are terminal nodes without outgoing edges to other nodes, except for potential self-loops. Cyclic states are confined to specific groups [19]. Additionally, feedback loops (also known as circuits) are crucial components of Boolean network analysis, playing a significant role in cellular regulation. Both positive and negative feedback loops are essential for maintaining homeostasis and enabling dynamic responses to stimuli [20]. Positive feedback loops are responsible for multistability, while negative feedback loops contribute to oscillations [21].

The integration of asynchronous updates allows for state modifications, reflecting the inherent non-deterministic nature of molecular networks [22,23,24,25,26]. This approach facilitates in silico perturbations such as gain-of-function (GoF) or loss-of-function (LoF) perturbations, enabling the precise examination of specific node influences on network dynamics and resulting phenotypes [18]. Specifically, GoF perturbations were simulated by setting the node to an active state (1) irrespective of its regulatory inputs, while LoF perturbations were simulated by fixing the node in an inactive state (0) under all conditions. These perturbations allow the exploration of how nodes such as p53, miR-34c, CricNOTCH1, and lncRNA MALAT1 affect cell fate outcomes and network stability. The results provide insights into the potential therapeutic implications of targeting these nodes in ferroptosis, apoptosis, and drug resistance pathways. Such methodologies support the exploration of the effects of nodes on network dynamics and subsequent phenotypic outcomes, thereby facilitating an investigation of the influences of specific nodes on network dynamics and resulting phenotypes [19].

## 3. Results

### 3.1. Dynamic Model and the Wild-Type Scenario Analysis

Ionizing radiation (IR) induces double-strand DNA breaks (DSBs) [27], which activate the ATM pathway and stimulate p53, either directly or by inhibiting MDM2 [28]. Our model includes essential p53 nodes—p53, p53_K (Ser46), p53_A (Ser15), and p53INP1—as described by Zhang et al. [29]. The primary p53 node interacts with Mdm2 to regulate p53 degradation [29], while activated p53 triggers phosphorylation, resulting in p53_A, which activates p21 to inhibit apoptosis and ferroptosis, and p53_K, which promotes ferroptosis and apoptosis through Bim [30], Bax [30], xCT [5], and Caspase 3 pathways [30]. The p53INP1 node manages the balance between these forms, affecting cell fates like apoptosis, ferroptosis, and senescence [29].

Additionally, p53-driven miR-34c targets Myc [12], SOX4 [15], Sirt1 [31], CDK 4/6CyclinD, and Bcl2 [32] to prevent lncRNA MALAT1 and CricNOTCH1 upregulation, reducing drug resistance by promoting apoptosis, ferroptosis, and senescence. By targeting Bcl2, Myc, and CDK4, miR-34c further induces apoptosis and senescence and enhances ferroptosis by suppressing Myc and MUC1, which are involved in xCT-mediated ferroptosis resistance. We integrated these insights into our model, highlighting therapeutic targets to counter resistance in non-small-cell lung cancer (NSCLC). For additional p53 functions, refer to Zhang et al. [29] By integrating these crucial biochemical insights into our model, we pinpointed promising therapeutic targets that could effectively combat cancer resistance, paving the way for more successful treatment options.

The model comprises 34 signaling components, integrating 3 non-coding RNAs: circRNA CricNOTCH1, lncRNA MALAT1, and miRNA miR-34c. The DNA damage response (DDR) is modeled as a binary input with “ON” and “OFF” states. Proteins are represented by white nodes, while circRNA and lncRNA are colored orange and violet, respectively, and miRNA is shown as a yellow node. The primary outputs, namely, proliferation, drug resistance, ferroptosis, apoptosis, and senescence, are clearly labeled. In total, the network contains 89 direct interactions, as visualized in Figure 1.

**Figure 2:**
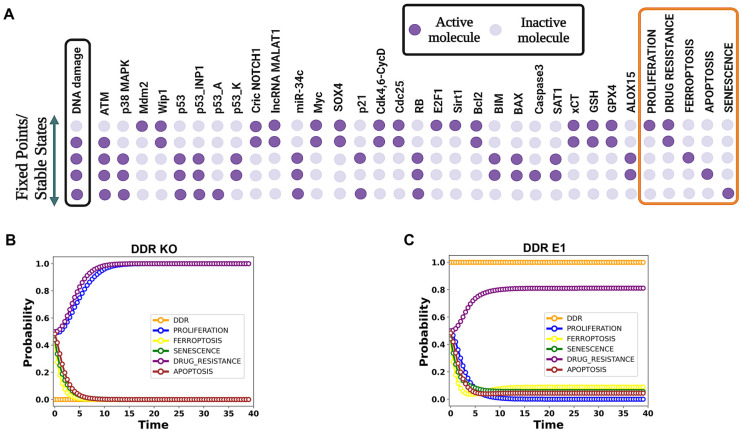
The dynamics of the wild-type case network, illustrating five stable states. (**A**) The leftmost column depicts the DNA damage levels highlighted in the black box, while the rightmost column presents the model outputs—proliferation, drug resistance, ferroptosis, apoptosis, and senescence—in the orange box. Each line represents an endpoint corresponding to the input. Light violet cells denote an inactivation, whereas dark violet cells denote activation. (**B**,**C**) Using MaBoSS simulations, we identified steady states under wild-type conditions with and without DNA damage. (**B**) For this analysis, DDR input was set to 0 (OFF), representing the absence of DNA damage. Under these conditions, the model predicted 100% proliferation and drug resistance, reflecting the baseline state of the system. (**C**) When DDR input was fully activated (probability = 1), representing maximal DNA damage, the model predicted distinct probabilities for cell fate outcomes: 81% drug resistance, 9% ferroptosis, 4% apoptosis, and 6% senescence. These outcomes sum to 100%, indicating the comprehensive distribution of cellular states in response to DNA damage. Detailed simulation data are available in Data S1.

In the wild-type (WT) system, five distinct steady states provide insights into the biological system’s behavior in the presence and absence of DNA damage (Figure 2A). The first state, proliferation, occurs when DNA damage (input) is “OFF” and is driven by CricNOTCH1, lncRNA MALAT1, Mdm2, Wip1, and oncogenes such as Myc and E2F1. When DNA damage is “ON”, the system shifts to one of four other states.

In the second state, drug resistance is activated through ATM, with CricNOTCH1, lncRNA MALAT1, Myc, E2F1, and Bcl2 inhibiting cell death mechanisms. The third state, ferroptosis, is induced by p53 (specifically p53-Ser46), miR-34c, and markers such as SAT1, ALOX15, BIM, and BAX, while caspase-3 remains suppressed. The fourth state, apoptosis, also triggered by p53-Ser46, activates pro-apoptotic proteins including BIM, BAX, and caspase-3. Finally, in the fifth state, senescence is induced through p53-Ser15 (p21), resulting in cell cycle arrest.

To capture the multifunctionality of p53, we utilize distinct p53 nodes—p53_K (p53_Killer for Ser46) and p53_A (p53_Arrest for Ser15)—as proposed by Zhang et al. [29]. The p53INP1 node acts as a crucial regulator, balancing p53_K and p53_A and ultimately influencing whether the cell undergoes death or enters a state of senescence.

Further MaBoSS simulations (Figure 2B,C) demonstrate the predicted steady-state outcomes under wild-type conditions. When DNA damage is OFF (DDR probability = 0), the model forecasts 100% proliferation and drug resistance, representing the baseline state of the system in the absence of DNA damage (Figure 2B). In contrast, when DNA damage is fully activated (DDR probability = 1), the model predicts distinct steady-state probabilities for various cell fate outcomes (Figure 2C). These include 81% drug resistance, 9% ferroptosis, 4% apoptosis, and 6% senescence. This distribution reflects the cellular response to maximal DNA damage, with all probabilities summing to 100%. Detailed simulation data are available in Data S1. For more details, see Figure 2.

### 3.2. Assessment of Dynamic Model Accuracy

To assess the model’s predictive capability, we performed gain-of-function (GoF) and loss-of-function (LoF) perturbations and verified the model’s ability to replicate experimental observations, ensuring that it accurately simulates the dynamic behavior of the biological system. Table 1 summarizes these validation findings, highlighting the robustness of the model’s predictions regarding individual nodes and their interactions. Key perturbations for xCT, CricNOTCH1, lncRNA MALAT1, and miR-34c were explored.

Our model, grounded in a WTp53 background, predicts the regulatory dynamics of ferroptosis, apoptosis, and drug resistance pathways under conditions that are consistent with functional p53. For example, in experimental systems where functional p53 is retained, xCT overexpression triggers Myc activation and the GSH/GPX4 pathway, leading to drug resistance and chemoresistance, as observed in oral squamous carcinoma cell lines (SAS, HSC2) [33]. Similarly, CricNOTCH1 overexpression inhibits miR-34c and reinforces drug resistance pathways in nasopharyngeal carcinoma (NPC) cells (SUNE1, SUNE2, and 6-10B) [12].

In osteosarcoma cells (MNNG/HOS, U2OS), lncRNA MALAT1 overexpression suppresses p53 and miR-34c, activating survival pathways through Myc and SOX4, which promote drug resistance [15]. Conversely, miR-34c overexpression promotes apoptosis and cell cycle arrest by targeting Bcl2 and Cdk4/6CyclinD pathways, as demonstrated in MG63, 143B, HOS, and Saos2 cells [32].

These perturbation experiments validate our model’s predictions within the context of functional p53 and provide critical insights into cell fate decisions, including ferroptosis and apoptosis, under DDR conditions. However, it is important to note that while these validation experiments focus on WTp53 contexts, cancers like oral squamous carcinoma, nasopharyngeal carcinoma, and osteosarcoma often exhibit high p53 mutation rates. This highlights a limitation of our model’s broader applicability to p53-mutant cancers.

Table 1 provides a detailed comparison of our model’s predictions with experimental observations. The consistent alignment underscores the reliability of our approach for studying cellular dynamics under WTp53 conditions.

### 3.3. CircNOTCH1 and lncRNA MALAT1 Independently Regulate Drug Resistance via miR-34c, with lncRNA MALAT1 Controlling xCT Expression in Cancer

To investigate whether CricNOTCH1 and/or lncRNA MALAT1 regulate xCT expression via miR-34c modulation, affecting cancer phenotypes such as drug resistance, we performed systematic gain-of-function (GoF) and loss-of-function (LoF) perturbations. We tested GoF for CricNOTCH1 and MALAT1 individually. Then, we examined combined GoF/LoF perturbations and finally performed dual knockdowns of both CricNOTCH1 and MALAT1.

**Figure 3:**
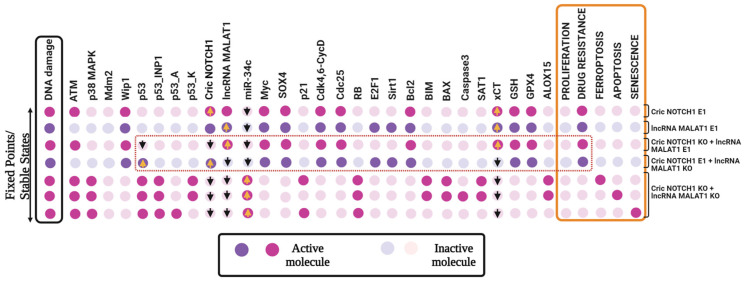
The gain or loss-of-function perturbations of the CricNOTCH1 and lncRNA MALAT1. The stable states identified for distinct scenarios were as follows: CricNOTCH1 E1, lncRNA MALAT1 E1, CricNOTCH1 KO + lncRNA MALAT1 E1, CricNOTCH1 E1 + lncRNA MALAT1 KO, CricNOTCH1 KO + lncRNA MALAT1 KO. E1 represents GoF and KO represents the LoF of the corresponding network element. The leftmost column shows DNA damage levels highlighted in black, and the rightmost column presents the model outputs, which are highlighted in orange: proliferation, drug resistance, ferroptosis, apoptosis, and senescence. Each line represents a single stable state corresponding to the input.

The results (Figure 3) show that the individual GoF of CricNOTCH1 or MALAT1 upregulates xCT expression, promoting drug resistance by suppressing miR-34c. In combinatorial settings, the GoF of CricNOTCH1 with LoF of MALAT1 did not induce xCT expression, while the opposite setup (GoF of MALAT1 with LoF of CricNOTCH1) triggered xCT expression and inhibited both p53 and miR-34c. Dual LoF perturbations of both CricNOTCH1 and MALAT1 reduced drug resistance and activated ferroptosis, apoptosis, and senescence.

These findings highlight that CricNOTCH1 indirectly influences drug resistance through miR-34c, while MALAT1 directly modulates xCT expression via p53 and miR-34c suppression. The dual inhibition of CricNOTCH1 and MALAT1 offers a promising approach with which to counteract drug resistance by promoting cell death pathways. See Figure 3 for detailed data.

### 3.4. The Role of p53 and ncRNAs in Modulating Ferroptosis, Apoptosis, and Senescence Pathways

To examine p53’s role in regulating ferroptosis, apoptosis, and senescence, we conducted a series of gain- and loss-of-function experiments on p53, miR-34c, CricNOTCH1, and lncRNA MALAT1. Our model differentiates between two phosphorylated forms of p53: p53_A (Ser15), promoting senescence through the p53/p21 pathway, and p53_K (Ser46), driving ferroptosis via the p53_K/SAT1 axis and apoptosis through the p53_K/caspase-3 pathway. Tang et al. [34] demonstrated that 20 µM Abivertinib (AC) induces 40% apoptosis and 60% ferroptosis in A549, MCF-7, and HeLa cells (all with wild-type p53), but did not assess senescence. This result contrasts with our model, where p53_A knockout maintained p53_K activity. This highlights the distinct roles of p53 forms in mediating cell fates.

We implemented various perturbations to assess these roles. First, we combined the p53_A knockout with miR-34c overexpression (p53_A KO + miR-34c E1) and p53_K knockout with miR-34c overexpression (p53_K KO + miR-34c E1). We also tested combined knockdowns of CricNOTCH1 and MALAT1 with either p53_A or p53_K knockouts. Additionally, we conducted individual perturbations, including the p53 knockout alone (p53 KO), miR-34c overexpression alone (miR-34c E1), and CricNOTCH1/MALAT1 knockdown alone (without p53 KO), to evaluate their specific contributions to ferroptosis, apoptosis, senescence, and drug resistance pathways.

The individual perturbation results showed less pronounced effects on cellular fates compared to the synergistic impacts observed in the combined perturbations. Notably, the MALAT1 knockout (MALAT1 KO) revealed the complete inhibition of proliferation and drug resistance (0%), consistent with MALAT1’s oncogenic role. Interestingly, ferroptosis (12%), apoptosis (7%), and senescence (7%) were not fully activated, suggesting that MALAT1 knockout partially destabilizes the network. These modest activations may reflect compensatory mechanisms among other regulatory elements, indicating that while MALAT1 is critical for promoting tumorigenesis, its absence does not entirely shift the system toward cell death pathways. Further investigation into the roles of upstream and downstream regulators is necessary to elucidate the precise dynamics at play. Additional individual perturbations, including simulation outputs for p53 KO, miR-34c E1, and CricNOTCH1 KO, further elucidate the network’s complexity and are provided in Supplementary Figure S1 and Data S1.

**Figure 4:**
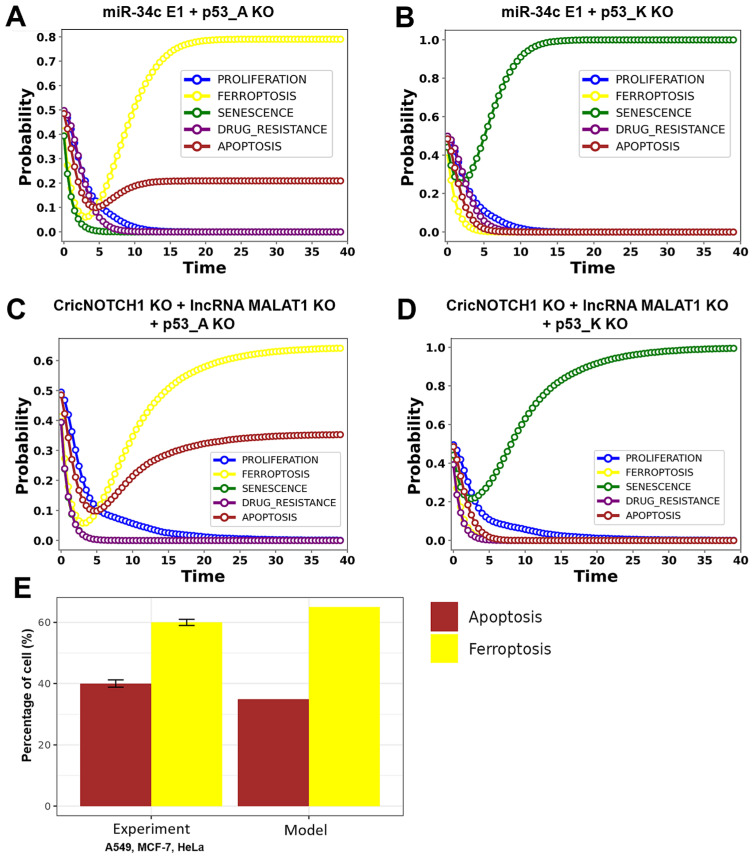
Dynamics of p53 and ncRNAs in DDR under the maximized DNA damage input. The DNA damage input (DDR) was initialized with a probability of 1, representing fully active conditions. (**A**) For the p53_K LoF + miR-34c GoF, we observed 100% senescence. (**B**) For the p53_A LoF + miR-34c GoF case, we observed 80% ferroptosis and 20% apoptosis. (**C**) Combined LoF CricNOTCH1 + LoF lncRNA MALAT1 + LoF p53_A led to 65% ferroptosis and 35% apoptosis. (**D**) Combined LoF CricNOTCH1 + LoF lncRNA MALAT1 + LoF p53_K resulted in 100% senescence, with no ferroptosis or apoptosis observed. (**E**) A comparison of the LoF CricNOTCH1, LoF lncRNA MALAT1, and LoF p53_A perturbations with in vitro observations. To enhance clarity and precision, fewer than 41 time steps are shown in the panel to highlight the differences among curves. The detailed simulation data can be found in Data S1.

Our results (Figure 4A) demonstrate that p53_A KO with miR-34c overexpression induces 80% ferroptosis and 20% apoptosis, while p53_K KO with miR-34c overexpression results in 100% senescence (Figure 4B). When knocking down both CricNOTCH1 and MALAT1 with p53_A KO, we observed 65% ferroptosis and 35% apoptosis (Figure 4C), whereas knockdown with p53_K KO led to 100% senescence (Figure 4D). Interestingly, the combined knockdown of CricNOTCH1, lncRNA MALAT1, and p53_A KO produced 35% apoptosis and 65% ferroptosis, closely matching the results of Tang et al. [34] for A549, MCF-7, and HeLa cells (see Figure 4E).

These findings confirm the distinct regulatory roles of p53_A and p53_K. Specifically, the knockdown of p53_K, combined with miR-34c overexpression or knockdowns of CricNOTCH1 and MALAT1, results in 100% senescence. In contrast, knockdown of p53_A with miR-34c overexpression induces 80% ferroptosis and 20% apoptosis, while combining p53_A knockdown with CricNOTCH1 and MALAT1 knockdowns triggers 65% ferroptosis and 35% apoptosis. Our model effectively captures these dual cell death mechanisms, providing valuable insights into potential therapeutic strategies for controlling cancer progression (Figure 4).

### 3.5. Exploring Feedback Loops in Drug Resistance and Cell Fate Decisions

Feedback loops (circuits) are critical for regulating cell fates, where positive loops stabilize cellular states and negative loops lead to oscillatory behaviors. Among the selected circuits, three key positive circuits stand out: CricNOTCH1/miR-34c/Myc, lncRNA MALAT1/miR-34c/Myc, and Myc/xCT. These were selected for their involvement in cell survival, apoptosis, ferroptosis, and drug resistance. Supplementary Table S2 outlines the positive and negative circuits, with these three circuits being particularly notable. These represent novel regulatory mechanisms predicted through our analysis, as no prior literature describes these specific interactions. The complex interactions and regulatory roles of these circuits are further elaborated in Table 2, offering deeper insights into their functions, which remain largely unexplored in existing studies.

**Figure 5:**
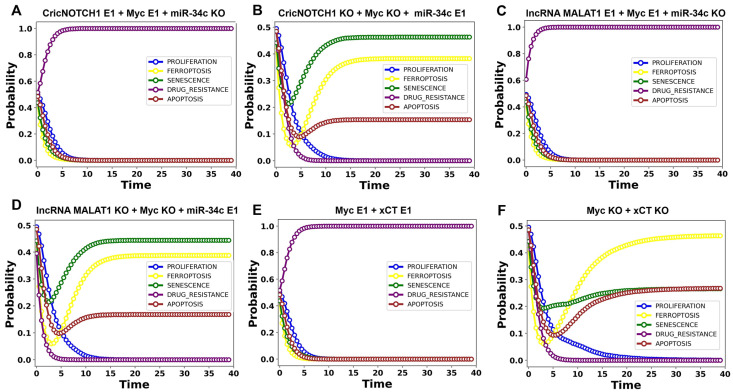
The dynamics of p53 and ncRNAs in DDR under maximized DNA damage input. Here, the DNA damage input (DDR) was initialized with a probability of 1, representing fully active conditions. (**A**) For the p53_K LoF + miR-34c GoF, we observed 100% senescence. (**B**) For the p53_A LoF + miR-34c GoF case, we observed 80% ferroptosis and 20% apoptosis. (**C**) The combined LoF CricNOTCH1 + LoF lncRNA MALAT1 + LoF p53_A led to 65% ferroptosis and 35% apoptosis. (**D**) The combined LoF CricNOTCH1 + LoF lncRNA MALAT1 + LoF p53_K resulted in 100% senescence, with no ferroptosis or apoptosis observed. (**E**) The combination of GoF Myc and GoF xCT led to 100% drug resistance. (**F**) The combination of LoF Myc and LoF xCT resulted in 47% ferroptosis, 27% apoptosis, and 26% senescence. To enhance clarity and precision, fewer than 41 time steps are shown in the panel to highlight the differences among curves. Detailed simulation data are available in Data S1.

Our perturbation analysis, conducted via MaBoss simulations, focused on tristability scenarios where ferroptosis, apoptosis, and senescence coexist (Figure 5). This method allowed us to differentiate between conditions associated with either elevated or reduced drug resistance. Specifically, scenarios where cell death phenotypes, ferroptosis, and apoptosis surpassed 70% were linked to diminished drug resistance, while outcomes below this threshold indicated increased resistance. These results, summarized in Figure 4, underscore miR-34c’s crucial role in regulating the balance between survival and cell death pathways across these regulatory circuits.

CricNOTCH1/miR-34c/Myc circuit: Knocking down miR-34c while overexpressing CricNOTCH1 and Myc shifted the system toward drug resistance (Figure 5A). Conversely, overexpressing miR-34c and knocking down CricNOTCH1 and Myc induced 39% ferroptosis, 16% apoptosis, and 45% senescence (Figure 5B), highlighting miR-34c’s ability to reprogram the circuit to promote cell death.

lncRNA MALAT1/miR-34c/Myc circuit: Suppressing miR-34c while overexpressing Myc and MALAT1 enhanced drug resistance (Figure 5C). However, the overexpression of miR-34c and the knockdown of Myc and MALAT1 induced 39% ferroptosis, 16% apoptosis, and 45% senescence (Figure 5D), demonstrating how miR-34c can counteract survival signals and shift the balance toward cell death.

Myc/xCT circuit: The co-overexpression of Myc and xCT promoted drug resistance, blocking ferroptosis, apoptosis, and senescence (Figure 5E). The knockdown of Myc and xCT reversed this phenotype, inducing 47% ferroptosis, 27% apoptosis, and 26% senescence (Figure 5F), underscoring the Myc/xCT axis as a potential therapeutic target to combat drug resistance.

Our findings highlight miR-34c’s pivotal role in regulating cell fates within the CricNOTCH1/miR-34c/Myc, MALAT1/miR-34c/Myc, and Myc/xCT circuits, impacting both drug resistance and cell death outcomes. Specifically, the overexpression of miR-34c in the CricNOTCH1/miR-34c/Myc and MALAT1/miR-34c/Myc circuits produces 45% senescence, with additional contributions to ferroptosis (39%) and apoptosis (16%), showing miR-34c’s capacity to drive senescence-dominant cell death. In the Myc/xCT circuit, targeting Myc and xCT induces a combined 74% cell death (47% ferroptosis and 27% apoptosis), indicating the robust suppression of drug resistance. These results demonstrate the therapeutic potential of manipulating miR-34c to shift cellular balance decisively toward cell death and reduced drug resistance (Figure 5).

## 4. Discussion

In this study, we present the first Boolean model to explore ferroptosis activation in cancer. The model integrates p53, miR-34c, CricNOTCH1, and lncRNA MALAT1 in the DDR, as shown in Figure 1. Our model identifies five distinct steady states: (1) proliferation with the upregulation of Mdm2, CricNOTCH1, and MALAT1; (2) drug resistance driven by ATM activation and involving CricNOTCH1, Myc, E2F1, and Bcl2; (3) ferroptosis initiated by p53_K (p53-Ser46), miR-34c, and ferroptosis markers SAT1 and ALOX15, with apoptosis suppressed; (4) apoptotic cell death involving p53_K, BIM, BAX, and caspase-3; and (5) senescence through p53_A (p53-Ser15)/p21 activation. Details are shown in Figure 2. Our WT case demonstrates how proteome diversity, exemplified by proteoform-specific activity of p53, shapes cell fate decisions in response to stress, highlighting the interplay between proteoforms and ncRNA networks in cancer.

We validated our model using gain- and loss-of-function (GoF/LoF) perturbations, confirming the roles of key regulators such as xCT, CricNOTCH1, lncRNA MALAT1, and miR-34c in ferroptosis, apoptosis, and drug resistance. xCT overexpression activated Myc and the GSH/GPX4 pathway, promoting drug resistance in oral squamous carcinoma [33]. CricNOTCH1 inhibited miR-34c, driving Myc expression and resistance in nasopharyngeal carcinoma. These findings provide a functional framework for understanding how key regulators like proteoforms and protein species mediate regulatory dynamics, influencing cancer cell fate. LncRNA MALAT1 repressed p53 and miR-34c, fostering proliferation in osteosarcoma [12]. miR-34c overexpression suppresses Bcl2, promotes apoptosis, and downregulates Cyclin D, leading to cell cycle arrest in MG63, 143B, HOS, and Saos2 cell lines [32]. These findings reveal critical insights into the regulatory dynamics of miR-34c, lncRNA MALAT1, and xCT in determining cancer cell fate. Table 1 shows more details. Additionally, we directly compared ferroptosis and apoptosis results from our model with in vitro studies using NSCLC (A549), breast cancer (MCF-7), and HeLa cells [34], as discussed in greater detail later.

Moreover, we reveal that CricNOTCH1 and lncRNA MALAT1 can influence xCT expression and drug resistance by inhibiting miR-34c and p53 (see Figure 3). Through GoF and LoF perturbation, we dissected their roles in cancer phenotypes. The GoF perturbation of Cric NOTCH1 or lncRNA MALAT1 increased xCT expression and drug resistance by downregulating miR-34c. However, combining CricNOTCH1 GoF with MALAT1 LoF increased p53 without xCT activation, while MALAT1 GoF with Cric NOTCH1 LoF activated xCT and suppressed both p53 and miR-34c. Dual LoF perturbation of both molecules inhibited drug resistance and promoted ferroptosis, apoptosis, and senescence, showing their complex interplay in cancer cell fate.

Supporting our findings, another member of miR-34 family (miR-34a-5p) was demonstrated to induce ferroptosis by targeting the Sirt1/peroxisome proliferator-activated receptor γ (PPARγ) coactivator-1α (Pgc-1α) axis and its downstream target Nrf2. Notably, Sirt1 is a well-established inhibitor of p53 activity. The overexpression of miR-34a activates p53, thereby promoting ferroptosis in MCF-7 cells [36]. In a parallel mechanism, the lncRNA MALAT1/miR-145-5p axis modulates ferroptosis through MUC1, a known inhibitor of p53, further reinforcing the pivotal role of p53 regulation in ferroptosis pathways in ectopic endometrial stromal cells [37]. Our results indicate that miR-34c targets Myc. This induces lncRNA MALAT1, ultimately activating p53, suppressing xCT, and initiating ferroptosis. This emphasizes the critical role of the miR-34c/Myc/MALAT1 axis in regulating ferroptosis and drug resistance through p53 signaling. Additionally, as Myc also upregulates xCT, its suppression by miR-34c reduces xCT levels, further promoting ferroptosis via the miR-34c/Myc/xCT axis.

Given the importance of proteoforms in modulating cell fate decisions, our study underscores the distinct contributions of p53 proteoforms, such as phosphorylated p53-Ser15 (p53_A) and p53-Ser46 (p53_K), to ferroptosis, apoptosis, and senescence. The differential PTMs of p53 [4], such as phosphorylation at Ser46, enable it to adopt specific proteoform states that govern unique cellular outcomes. Proteoforms like phosphorylated p53-Ser46 contribute to ferroptosis and apoptosis [38], while others may inhibit cell death by activating pathways like p21 [39], demonstrating the complexity of proteome regulation. Proteoforms, arising from PTMs and genetic variations, exhibit unique functional roles that critically influence cancer cell fate decisions [4]. This proteoform diversity accounts for distinct cellular outcomes, including ferroptosis, apoptosis, and senescence. As recent studies suggest that p53 can both trigger and inhibit ferroptosis, particularly through the p53/p21 pathway [40,41], Tarangelo et al. [40] found that the p53-p21 axis helps cancer cells to manage metabolic stress from cystine deprivation, inhibiting ferroptotic cell death in U2OS, A549, T98G, H1299, ACHN, and CAKI-1 cell lines. Similarly, Kuganesan et al. [41] showed that high p53 level enhance ferroptosis, while p53/p21 pathway acts as a suppressor ferroptosis in TR9-7, HT1080-LXSN, HT1080-GSE56, NARF2 pBpuro cell lines.

To further investigate this dual regulation, we conducted a series of perturbation experiments (see Figure 5). These perturbations involved knocking down specific p53 variants (p53_A KO + miR-34c E1 and p53_K KO + miR-34c E1) and assessing their effects. Additionally, we examined the combined knockdown of CricNOTCH1 and lncRNA MALAT1, alongside individual knockdowns of p53_A and p53_K, to determine their regulatory roles.

Our analysis aligns with findings by Tang et al. [34], who reported that administering 20 µM Abivertinib (AC) for 6 h led to 40% apoptosis and 60% ferroptosis in A549, MCF-7, and HeLa cells, primarily through the Bim and Bax pathways. Bim and Bax [30], known regulators of cell death, are p53-induced, with recent evidence suggesting p53 also triggers Bim [42,43], which aligns with our findings on p53’s role in apoptosis and ferroptosis. This comparison with our p53_A knockout (p53_A KO) model, wherein p53_K remains active, supports the notion that p53 isoforms mediate diverse cellular outcomes.

Although Tang et al.’s study [34] did not address cell cycle arrest or senescence, it is important to note that all three cell lines (A549, MCF-7, and HeLa) possess functional wild-type p53 (WTp53). Our findings suggest that full p53 activation with miR-34c can result in various outcomes, including ferroptosis, apoptosis, and senescence. Notably, the knockdown of p53_A while overexpressing miR-34c completely abolished senescence, favoring an 80% ferroptosis and 20% apoptosis ratio through p53_K, thereby reinforcing the notion that the p53/p21 pathway serves to inhibit ferroptosis. In contrast, the combined knockdown of CricNOTCH1 and lncRNA MALAT1 alongside p53_A KO resulted in 35% apoptosis and 65% ferroptosis (Figure 4), closely resembling the apoptosis/ferroptosis phenotypes observed by Tang et al. [34]. This alignment of our findings with those of Tang et al. [34], Tarangelo et al. [40], and Kuganesan et al. [41] underscores the efficacy of our dynamic model in elucidating the dual mechanisms of cell death governed by p53 and ncRNAs. In particular, the p53/SAT1 [6] and BIM/BAX [34] pathways facilitate ferroptosis independently of caspase-3, as well as caspase-3-dependent apoptosis [34], while the p53/p21 axis serves to inhibit these cell death processes [40,41].

Furthermore, the cancers used in our broader validation, such as oral squamous cell carcinoma, nasopharyngeal carcinoma, and osteosarcoma, often harbor a high prevalence of p53 mutations. Despite this, the alignment of our model with experimental data reflects scenarios where WTp53 is functionally active. Future work could extend the model to integrate mutant p53 dynamics, which would enhance its applicability, particularly in contexts of drug resistance and p53-mutant cancers.

In addition, we investigated several positive circuits that play essential roles in cancer cell survival, drug resistance, and cell death mechanisms such as apoptosis, ferroptosis, and senescence. Circuits, particularly positive circuits, are critical for maintaining cellular homeostasis by amplifying or suppressing molecular signals. These circuits often involve double-negative or double-positive interactions, and their dysregulation can lead to cancer progression by stabilizing oncogenic states. Our results highlight the importance of three novel positive circuits—CricNOTCH1/miR-34c/Myc, lncRNA MALAT1/miR-34a/Myc, and Myc/xCT—which have not been extensively explored in prior studies but are predicted to play significant roles in regulating cancer cell fate decisions (see Table 2 and Figure 5). Importantly, these circuits are shaped by proteome complexity, where proteoforms of Myc dynamically influence the feedback loops, either amplifying oncogenic signals or promoting pathways like ferroptosis and apoptosis.

The CricNOTCH1/miR-34c/Myc and MALAT1/miR-34c/Myc circuits highlight miR-34c as a critical suppressor of Myc, influencing cellular outcomes. The downregulation of miR-34c via CricNOTCH1 or MALAT1 overexpression promotes drug resistance by inhibiting ferroptosis and apoptosis, consistent with its role as a tumor suppressor. The overexpression of miR-34c, on the other hand, shifts the balance toward cell death, inducing ferroptosis, apoptosis, and notably, senescence (45%), highlighting its dual role in regulating cell fate.

The Myc/xCT circuit, however, reveals a distinct mechanism of drug resistance. The co-overexpression of Myc and xCT blocks cell death, whereas knocking both down increases susceptibility to 74% cell death, primarily through ferroptosis and apoptosis. This suggests that targeting the Myc/xCT axis could effectively reduce drug resistance. Together, these findings underscore miR-34c’s therapeutic potential in shifting the balance toward cell death, while also positioning the Myc/xCT pathway as a promising target for overcoming resistance in precision oncology. Figure 5 displays more details.

Our results demonstrate that miR-34c acts as a central regulator within intricate feedback loops, significantly influencing cell fate decisions. The regulatory circuits involving CricNOTCH1, lncRNA MALAT1, miR-34c, Myc, and xCT elucidate how cancer cells evade cell death and resist therapy. Given the novelty and importance of these feedback loops in promoting cancer survival, targeting them presents promising therapeutic opportunities, particularly for overcoming drug resistance in cancer.

**Figure 6:**
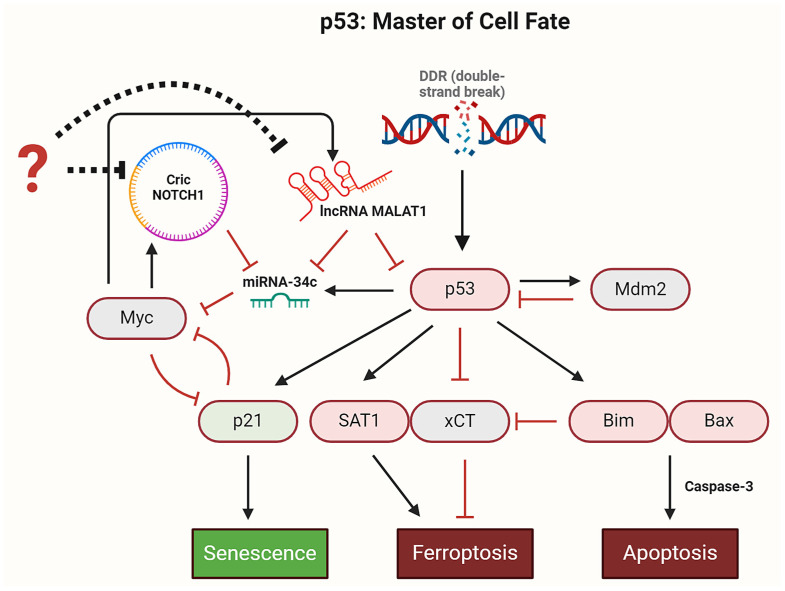
p53 Dynamics in the DDR. Upon DNA damage, p53 is activated and induces Mdm2 expression, which subsequently inhibits p53 activity. Once activated, p53 regulates several critical signaling pathways: it induces p21 to trigger senescence, activates the BIM/BAX axis to initiate apoptosis, and promotes ferroptosis by either directly inhibiting xCT or inducing SAT1 expression. Additionally, p53 activates miR-34c, which suppresses Myc, a potent inducer of CricNOTCH1 and lncRNA MALAT1. By inhibiting Myc, miR-34c reduces the expression of CricNOTCH1 and lncRNA MALAT1. Interestingly, lncRNA MALAT1 directly inhibits p53. Therefore, miR-34c promotes sustained p53 activation by inhibiting Myc, which in turn diminishes MALAT1’s inhibitory effect on p53. In addition, inhibitors (indicated by question mark) of CricNOTCH1 and lncRNA MALAT1 trigger p53-mediated signaling pathways such as ferroptosis, apoptosis and senescence in DDR. Black arrows represent activation, hammerhead arrows represent inhibition, and dotted hammerhead arrows indicate indirect inhibition.

Building on these findings, future research should focus on integrating multi-omics data, such as single-cell proteomics and phosphoproteomics, to better characterize the diverse landscape of proteoforms and their role in shaping regulatory networks. These studies will help to validate our computational predictions and deepen our understanding of how proteome-driven dynamics influence cancer progression. Ultimately, such insights will enable the identification of proteoform-specific targets, paving the way for personalized treatment strategies that harness proteome diversity to overcome drug resistance and promote targeted cell death. Despite the complexity of these regulatory networks, it is clear that p53 plays a central role as a master regulator of cell fate decisions in DDR, as illustrated in Figure 6.

## 5. Conclusions

In summary, this study provides a detailed exploration of the complex interplay between ferroptosis, apoptosis, senescence, and drug resistance in cancer, focusing on the pivotal roles of p53, miR-34c, CricNOTCH1, and lncRNA MALAT1 within the DDR framework. Our findings demonstrate how p53, in conjunction with miR-34c, orchestrates the dual induction of ferroptosis and apoptosis while simultaneously suppressing drug resistance mechanisms, revealing a finely tuned balance critical for tumor suppression. The proteoform states of p53, particularly in relation to PTMs, further contribute to the complexity of cellular responses, enabling the dynamic regulation of cell fate decisions. Moreover, we identify a previously unrecognized regulatory axis involving CricNOTCH1 and lncRNA MALAT1, underscoring the regulatory importance of ncRNAs in modulating cellular stress responses and therapeutic outcomes. The discovery of positive feedback loops involving CricNOTCH1, lncRNA MALAT1, miR-34c, Myc, and xCT further enhances our understanding of proteomic complexity, particularly in the context of cancer cell survival and therapy resistance. While this study primarily focuses on specific carcinoma types, the molecular mechanisms proposed here, particularly in relation to proteoform dynamics, are adaptable to a broad range of cancers, including NSCLC and breast cancer. The insights gained from the proteomic complexity of p53 and its interactions with ncRNAs provide valuable directions for future therapeutic strategies aimed at reactivating p53-mediated tumor suppression while overcoming drug resistance. Future research should focus on the clinical validation of these regulatory circuits, further elucidating the proteoform-dependent regulation of these pathways and exploring their potential in developing more precise, targeted cancer therapies.

## Figures and Tables

**Table 1:** Comprehensive validation. We compared the effects of overexpression (E1), denoting gain-of-function (GoF) perturbations, and knockdown (KO), indicating loss-of-function (LoF) perturbations, on known experimental observations in breast cancer.

Consistency Between the Dynamic Model Predictions and Known Experimental Observations					
Stimulus/Perturbations	Model Response	Experimental Observations	Agreement	Cell Lines	Ref.
xCT E1	xCT overexpression induces Myc activity and the GSH/GPX4 pathway, promoting drug resistance.	xCT overexpression increases GSH/GPX4 activity, promoting chemoresistance, therapy resistance, and EMT.	Yes	SAS and HSC2 (oral squamous carcinoma)	[33]
CricNOTCH1 E1	CricNOTCH1 overexpression inhibits miR-34c, increasing Myc, xCT/GSH/GPX4 axis, and lncRNA MALAT1, promoting drug resistance.	CricNOTCH1 acts as a competitive endogenous RNA (ceRNA), modulating Myc expression by sponging miR-34c and promoting an aggressive malignant phenotype.	Yes	NPC cell lines (SUNE1, SUNE2, and 6-10B) (nasopharyngeal carcinoma)	[12]
In DDR, lncRNA MALAT1 E1	lncRNA MALAT1 overexpression inhibits p53 and miR-34c, activating survival pathways by inducing Myc and SOX4, which promote drug resistance.	MALAT1 overexpression inhibits miR-34c, thereby activating SOX4 and promoting EMT and proliferation.	Yes	MNNG/HOS, Saos-2, U2OS, MG-63 (osteosarcoma)	[15]
miR-34c E1	miR-34c overexpression inhibits Bcl2 induces apoptosis.	Overexpression of miR-34c, promoting caspase-3/9-dependent apoptosis.	Yes	MG63, 143B, HOS, and Saos2 (Osteosarcoma)	[32]
miR-34c E1	miR-34c overexpression inhibits cdk4, 6, Cyclin D block the cell cycle.	Overexpression of miR-34c, promoting cell cycle arrest by targeting Cyclin D1.	Yes	MG63, 143B, HOS, and Saos2 (Osteosarcoma)	[32]

**Table 2:** Experimental observations of predicted positive circuit: detailed mechanisms and interactions within CricNOTCH1/miR-34c/Myc (double-negative) circuit, the lncRNAMALAT1/miR-34a/Myc (double-negative) circuit, and Myc/xCT positive circuit.

Positive Circuit	Circuit Elements	Targets	Interaction Type	Ref.	Mechanism Involved
CricNOTCH1/miR-34c/Myc	CricNOTCH1	miR-34c	ceRNA inhibition	[12]	CricNOTCH1 sequesters miR-34c, preventing it from targeting its mRNA targets, leading to the increased expression of genes that promote cell survival.
	miR-34c	Myc	Direct inhibition	[12]	miR-34c binds to Myc mRNA, inhibiting its translation and reducing Myc-driven processes such as cell growth and proliferation.
	Myc	CricNOTCH1	Transcriptional activation	[12]	Myc binds to the CricNOTCH1 promoter, activating its transcription, which leads to increased levels of CricNOTCH1 and the further inhibition of miR-34c.
lncRNA MALAT1/miR-34c/Myc	lncRNA MALAT1	miR-34c	ceRNA inhibition	[15]	MALAT1 inhibits miR-34c by acting as a ceRNA, sequestering miR-34c and preventing it from targeting its mRNAs.
	miR-34c	Myc	Direct inhibition	[35]	miR-34c suppresses Myc by binding to its mRNA, inhibiting translation and reducing Myc-driven processes like DNA synthesis.
	Myc	lncRNA MALAT1	Transcriptional Activation	[13]	Myc binds to the MALAT1 promoter, directly activating its transcription.
Myc/xCT	Myc	xCT	Transcriptional activation	[7,8]	Myc directly induces xCT expression, enhancing glutamate export and contributing to ferroptosis resistance.
	xCT	Myc	Transcriptional activation	[9,10]	xCT promotes c-Myc expression by increasing intracellular cysteine levels, enhancing Myc expression and activity.

## Data Availability

The model code is available in a publicly accessible GitHub repository, which can be accessed through the following link: https://github.com/GuptaShan/A-Model-of-p53-and-ncRNAs-in-Ferroptosis (accessed on 15 January 2025).

## Supplementary Materials

The following supporting information can be downloaded at: https://www.mdpi.com/article/10.3390/proteomes13010006/s1. Table S1: Logical Rules Governing Node Activity and Full Node Names. Data S1: Detailed Simulation Data for Model Results. Figure S1: Effects of Individual Molecule Perturbations on Key Regulators Under DNA Damage. Table S2: Boolean Network Circuits and Their Experimental Validation. References [44,45,46,47,48,49,50,51,52,53,54,55,56,57,58,59,60,61,62,63,64,65,66,67,68,69,70,71,72,73,74,75,76,77,78,79,80,81,82,83,84,85,86,87,88,89,90,91,92,93] are cited in the supplementary materials.
