# Supplementary material for: DNA Damage-Induced Ferroptosis: A Boolean Model Regulating p53 and Non-Coding RNAs in Drug Resistance
Source: Proteomes. 2025 Jan 20;13(1):6. doi: 10.3390/proteomes13010006 (PMC11755436; doi:10.3390/proteomes13010006)
Supplement: Supplementary file 1 [file proteomes-13-00006-s001.zip › Table S2.pdf]

Supplementary Table S2

Table S2: Boolean network functional circuits and corresponding experimental observations. Cases not yet studied experimentally are marked as "Predicted".

Positive Circuits	References
E2F1/Myc	[1]
p21/Caspase3	[2]
CricNOTCH1/miR-34c/Myc	Predicted
LncRNAMALAT1/miR-34c /Myc	Predicted
xCT/Myc	Predicted
p53_A/p53_K	[3]
p21/Myc	[4]
E2F1/ATM	[5]
**Negative Circuits**	**References**
p53/Mdm2	[6]
p53_A/p53_INP1	[3]
E2F1/Sirt1	[7]

References:

[1] H.A. Coller, J.J. Forman, A. Legesse-Miller, "Myc'ed messages": myc induces transcription of E2F1 while inhibiting its translation via a microRNA polycistron, PLoS Genet 3 (2007) e146. https://doi.org/10.1371/journal.pgen.0030146.

[2] Y. Zhang, N. Fujita, T. Tsuruo, Caspase-mediated cleavage of p21Waf1/Cip1 converts cancer cells from growth arrest to undergoing apoptosis, Oncogene 18 (1999) 1131–1138. https://doi.org/10.1038/sj.onc.1202426.

[3] X.-P. Zhang, F. Liu, W. Wang, Two-phase dynamics of p53 in the DNA damage response, Proc Natl Acad Sci U S A 108 (2011) 8990–8995. https://doi.org/10.1073/pnas.1100600108.

[4] R.U. Jänicke, D. Sohn, F. Essmann, K. Schulze-Osthoff, The multiple battles fought by anti-apoptotic p21, Cell Cycle 6 (2007) 407–413. https://doi.org/10.4161/cc.6.4.3855.

[5] W.C. Lin, F.T. Lin, J.R. Nevins, Selective induction of E2F1 in response to DNA damage, mediated by ATM-dependent phosphorylation, Genes Dev 15 (2001) 1833–1844.

[6] R.L. Bar-Or, R. Maya, L.A. Segel, U. Alon, A.J. Levine, M. Oren, Generation of oscillations by the p53-Mdm2 feedback loop: A theoretical and experimental study, PNAS 97 (2000) 11250–11255. https://doi.org/10.1073/pnas.210171597.

[7] C. Wang, L. Chen, X. Hou, Z. Li, N. Kabra, Y. Ma, S. Nemoto, T. Finkel, W. Gu, W.D. Cress, others, Interactions between E2F1 and SirT1 regulate apoptotic response to DNA damage, Nature Cell Biology 8 (2006) 1025–1031.
