# Supplementary material for: DNA Damage-Induced Ferroptosis: A Boolean Model Regulating p53 and Non-Coding RNAs in Drug Resistance
Source: Proteomes. 2025 Jan 20;13(1):6. doi: 10.3390/proteomes13010006 (PMC11755436; doi:10.3390/proteomes13010006)
Supplement: Supplementary file 1 [file proteomes-13-00006-s001.zip › Figure S1.pdf]

Supplementary Figure S1

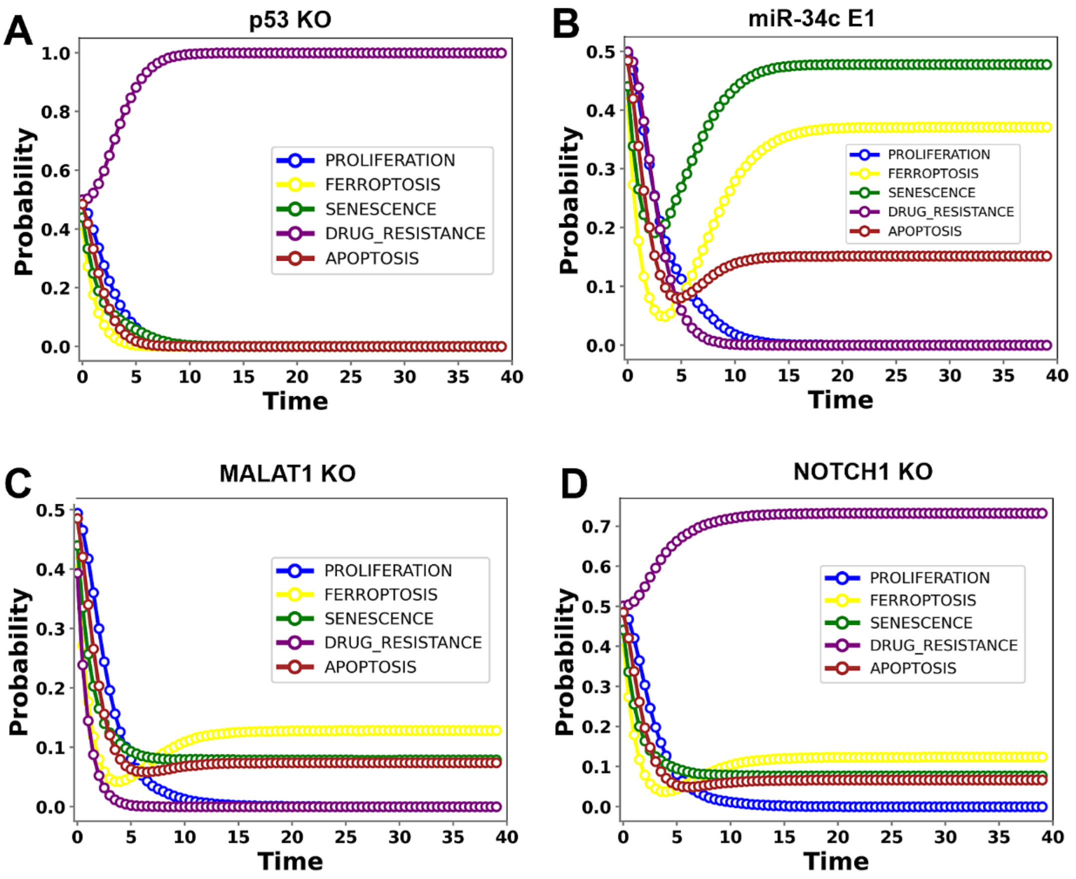

Figure S1. Effects of individual perturbations on p53, miR-34c, lncRNA MALAT1, and CricNOTCH1 under fully activated DNA damage conditions. Simulations were performed with the DNA Damage Response (DDR) input was initialized with a probability of 1, representing fully active conditions. (A) Loss-of-function (LoF) of p53 resulted in 100% drug resistance, emphasizing its critical role in mediating therapeutic responses. (B) Gain-of-function (GoF) of miR-34c led to 37% ferroptosis, 15% apoptosis, and 48% senescence, demonstrating its regulatory impact on cell fate decisions. (C) LoF of lncRNA MALAT1 yielded 12% ferroptosis, 7% apoptosis, and 7% senescence, with complete abrogation of drug resistance (0%), highlighting its oncogenic role. (D) LoF of CricNOTCH1 resulted in 73% drug resistance, 12% ferroptosis, 7% apoptosis, and 8% senescence, underscoring its involvement in tumor resilience. Fewer than 41-time steps are shown to enhance clarity and focus on the divergence among simulation curves. Detailed simulation data and time-course results are available in S1 Data.
